# Serum Lipids Alterations in Patients Under Systemic JAK Inhibitors Treatments in Dermatology: Clinical Aspects and Management

**DOI:** 10.3390/medicina61010054

**Published:** 2025-01-01

**Authors:** Giovanni Paolino, Mario Valenti, Andrea Carugno, Matteo Bianco, Dario Didona, Matteo Riccardo Di Nicola, Pier Luigi Acutis, Carmen Cantisani, Vittoria Giulia Bianchi, Nicola Zerbinati, Alessandra Narcisi, Antonio Costanzo, Santo Raffaele Mercuri

**Affiliations:** 1 Unit of Dermatology and Cosmetology, IRCCS Ospedale San Raffaele, 20132 Milan, Italy; paolino.giovanni@hsr.it (G.P.); bianchi.vittoriagiulia@hsr.it (V.G.B.); mercuri.santoraffaele@hsr.it (S.R.M.); 2 Faculty of Medicine and Surgery, Vita-Salute San Raffaele University, 20132 Milan, Italy; 3 Dermatology Unit, IRCCS Humanitas Research Hospital, 20089 Rozzano, Italy; valenti.mario.92@gmail.com (M.V.); matteo.bianco@humanitas.it (M.B.); alessandra.narcisi@humanitas.it (A.N.); antonio.costanzo@hunimed.eu (A.C.); 4 Department of Biomedical Sciences, Humanitas University, Pieve Emanuele, 20089 Milan, Italy; 5 Department of Medicine and Surgery, University of Insubria, 21100 Varese, Italy; 6 Rare Skin Diseases Center, Istituto Dermopatico dell’Immacolata, IDI-IRCCS, 00167 Rome, Italy; dario.didona@gmail.com; 7 Istituto Zooprofilattico Sperimentale del Piemonte, Liguria e Valle d’Aosta, 10154 Turin, Italy; pierluigi.acutis@izsplv.it; 8 Dermatologic Unit, Department of Clinical Internal, Anesthesiological and Cardiovascular Sciences, La Sapienza University of Rome, 00185 Rome, Italy; carmencantisanister@gmail.com; 9 Department of Medicine and Innovation Technology (DiMIT), University of Insubria, 21100 Varese, Italy; nzerbinati@centro-medico.it

**Keywords:** atopic dermatitis, alopecia areata, vitiligo, cardiovascular risk, dermatological treatment, dyslipidemia, JAK inhibitors, JAK-STAT signaling, janus kinase, lipid metabolism, serum lipids

## Abstract

*Background and Objectives*: Janus kinase inhibitors (JAKi) have significantly advanced the treatment of various dermatological conditions by modulating the JAK-STAT signalling pathway. While these inhibitors have proven effective, they also pose challenges due to associated increase in serum lipid levels and relative potential cardiovascular risks. This perspective work aims to discuss the implications of these lipid alterations proposing management strategies for patients with dermatological disorders under JAKi treatments. *Materials and Methods*: This manuscript reviews existing and recent literature on the metabolic effects of JAKi, particularly focusing on their impact on lipid profiles in patients treated for dermatological diseases. *Results*: JAK inhibitors are consistently associated with an increase in both LDL and HDL levels shortly after treatment initiation, which tend to stabilise over time. Despite these changes, there is no clear evidence linking these alterations to an increased risk of major adverse cardiovascular events (MACE), indicating a complex interaction between lipid metabolism and JAK-STAT signalling. *Conclusions*: Although JAKi may induce lipid changes in patients, raising concerns, especially in ones with existing cardiovascular risks, currently there is no proven link to increased MACE in this population. Monitoring lipid levels, alongside lifestyle modifications and possible statin use, can manage these effects without the need to stopping treatment.

## 1. Introduction

The Janus kinase/signal transducer and activator of transcription (JAK/STAT) signaling pathway involves families of molecules associated with the intracellular domains of receptors for various cytokines and growth factors, facilitating signal transmission to the cell nucleus [1,2]. The JAK-STAT pathway plays a pivotal role in several cellular functions, including mitosis, differentiation, apoptosis, hematopoiesis, immune system (both innate and adaptive), and exocrine gland activity. Through some extracellular mediators, it regulates the expression of specific genes that govern key cellular functions [1]. Besides, hypoxia, ultraviolet radiation, endotoxin exposure, and oxidative and hyperosmolar stress can be modulated through the activation of this pathway, resulting in distinct cellular responses. These responses can, in turn, trigger inflammatory reactions, contributing to the onset of diseases such as atopic dermatitis, psoriasis, alopecia areata and vitiligo. Blocking this signal transduction pathway can therefore reduce or modulate inflammation in these conditions, as evidenced by the increasing clinical use of JAK inhibitors (JAKi). While the literature extensively explores JAKi and their impact on lipid metabolism, we identified a gap in the availability of a concise and practical guide tailored for dermatologists seeking quick reference in clinical practice. The aim of this perspective article is to evaluate the main pathophysiological aspects of dyslipidemia development associated with JAK inhibitors in dermatology and to discuss the relative management strategies, given the growing prevalence of these agents in daily clinical practice. However, before addressing these points, it is essential to understand the fundamental physiological aspects of this significant signaling pathway.

### 1.1. JAKs and STATs and Their Structures

JAK proteins are characterised by four domains and seven regions (JH1-JH7); among these, JH1 domain (active kinase domain) is involved in phosphate transfer and subsequent nuclear activation, making it the primary target for JAK inhibitors. However, since JH1 shows a high degree of homology with other tyrosine kinases (TYKs), achieving selectivity for JAK inhibitors is challenging [1]. Contrariwise, JH2 domain (pseudo-kinase domain) acts more as a regulatory region, suppressing ligand-independent kinase activity by interacting directly with JH1 [1]. At the same time, JH5, JH6, and JH7 regions (collectively known as the FERM domains) are essential for the interaction between JAKs and specific receptors [1]. Finally, JH3 and JH4 stabilise the conformation of the JAK FERM, appearing to be essential for JAK protein function [3].

### 1.2. JAK–STAT Signaling

**Figure 1:**
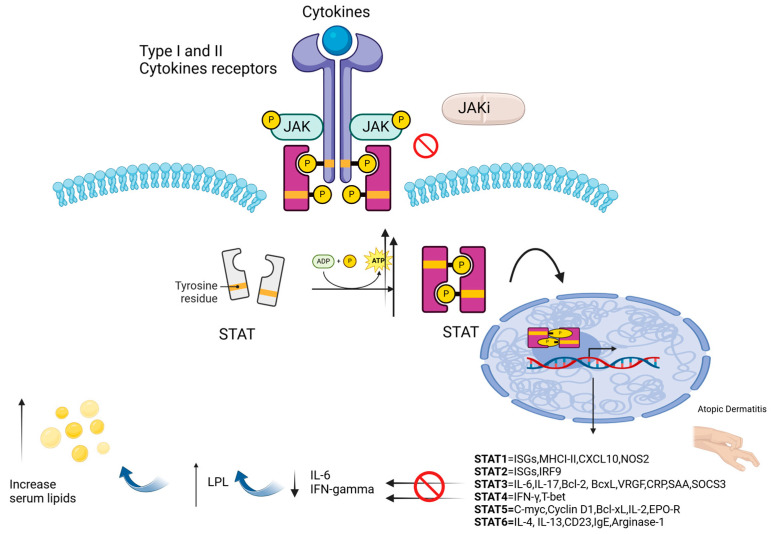
Mechanism of action of JAK-inhibitors. Once STAT dimers enter the nucleus, they act as transcription factors by binding to specific DNA sequences (such as Gamma-Activated Sites or Interferon-Stimulated Response Elements), located in the promoters of target genes. The specific genes transcribed depend on the type of STAT activated, the cytokine or ligand involved, and the cell type. STAT1 primarily transcribes genes involved in antiviral responses, such as interferon-stimulated genes (ISGs) like MX1, OAS1, PKR, and IRF1. It also induces the expression of MHC class I and II molecules, enhancing antigen presentation, and IP-10 (CXCL10), which recruits immune cells like T lymphocytes and NK cells to sites of infection or inflammation. STAT2, often in combination with STAT1 and IRF9, transcribes additional ISGs that enhance antiviral defenses, like ISG15 and other resistance-related genes. STAT3 is involved in promoting cell survival and inflammatory responses. It induces the transcription of anti-apoptotic genes such as Bcl-2 and Bcl-xL, as well as factors like vascular epithelial growth factor (VEGF), which stimulates angiogenesis. STAT3 also upregulates acute-phase proteins, including CRP (C-reactive protein) and serum amyloid A (SAA), which are important during systemic inflammation. STAT4 is key in driving the differentiation of T cells into the Th1 phenotype. It promotes the transcription of IFN-γ, a central cytokine in cellular immunity, and T-bet, a transcription factor that further reinforces Th1 responses. STAT5 plays a pivotal role in regulating cell growth, hematopoiesis, and adaptive immunity. It transcribes genes such as C-myc, which promotes cell proliferation, Cyclin D1, which regulates the cell cycle, and Bcl-xL, which supports cell survival. STAT5 also enhances IL-2 signaling, crucial for T cell function, and contributes to erythropoiesis by promoting the expression of the erythropoietin receptor (EPO-R). STAT6 is central to Th2 immune responses and allergic reactions. It transcribes genes like IL-4 and IL-13, which amplify Th2 polarization, and promotes IgE production through the upregulation of CD23. It also induces Arginase-1, involved in tissue repair and modulation of inflammation. Increased levels of serum lipids in patients under JAK inhibitors treatment (JAKi). JAKi by inhibiting interleukin (IL)-6 and Interfeton (IFN)-gamma increase lipoprotein lipase (LPL) activity leading to an increase of serum lipids levels. Created in BioRender. Paolino, G. (2025) https://BioRender.com/c47u896 (accessed on 23 December 2024)”.

The JAK/STAT pathway is activated when type I and type II cytokines bind to their receptors, which are characterised by different chains. This binding activates JAKs, which phosphorylate themselves and their relative receptors, creating binding sites for STAT proteins. Finally, these active STATs move into the nucleus to regulate gene expression [1]. There are four types of JAKs: JAK1, JAK2, JAK3, and TYK2 [1,3,4,5]. While, regarding STAT proteins, there are seven types: STAT1, STAT2, STAT3, STAT4, STAT5A, STAT5B, and STAT6. When cytokine receptors activate JAKs, STATs are phosphorylated and form dimers or tetramers, undergoing both tyrosine and serine phosphorylation by other kinases [1]. Specifically, serine phosphorylation enhances gene activation and supports energy production in mitochondria. Even without phosphorylation, STATs can still dimerize and regulate genes (Figure 1). It is interesting that any alteration in this pathway can lead to different diseases, as follows: chronic infections (e.g., viral diseases, mycological diseases, bacterial diseases) in case of deficit STAT1; psoriasis, atopic dermatitis, alopecia areata and vitiligo in case of STAT1 hyper-activation. RNA viruses infections (e.g., influenza virus) in case of deficit STAT2. Malignancies (lung cancer, breast cancer, leukemia, lymphomas) and autoimmune and/or chronic inflammatory diseases (e.g., lupus erythematosus, psoriasis, atopic dermatitis, vitiligo, alopecia areata, rheumatoid arthritis) diseases in case of STAT3 hyper-activation; hyper-IgE syndrome and chronic bacterial or mycological infections in case of STAT3 deficit. Reduced Th1 response and relative predisposition to viral and bacteriological infections and autoimmune diseases (e.g., lupus erythematosus) in case of deficit STAT4. Chronic myeloid leukemia in STAT 5 hyper-activation; prolactin production alteration in case of STAT5A/B deficit. Asthma, atopic dermatitis in case of hyper-activation STAT6 and Th2 deficit in case of downregulation of STAT6 [6]. Table 1 summarises the main cytokines involved in the JAK/STAT pathway.

### 1.3. JAK Inhibitors

Our discussion so far highlights the essential role of JAKs and STATs in maintaining immune system balance, supporting the targeting of JAK–STAT signalling for treating autoimmune and inflammatory conditions. Since 1990s, JAK inhibitors have emerged as a means of controlling inflammation through JAK inhibition. Currently available JAK inhibitors work by blocking the ATP-binding site in the JH1 domain through non-covalent interactions. However, due to the structural similarity of this site with other tyrosine kinases, it has been necessary to make this mechanism of action highly selective [3]. Various JAK inhibitors with reasonable specificity have been developed, and pan-JAK inhibitors (targeting multiple JAKs) have shown effectiveness with manageable side effects; however, how much the selectivity of a JAK inhibitor can enhance its mechanism of action remains unclear [3], (Figure 1).

The development of small molecules that inhibit JAK activities have broadened the therapeutic landscape for many chronic inflammatory and autoimmune diseases. To date, twelve JAK inhibitors have received clinical approval: abrocitinib, baricitinib, delgocitinib, deucravacitinib, fedratinib, filgotinib, oclacitinib, pacritinib, peficitinib, ruxolitinib, tofacitinib, and upadacitinib. Among these, abrocitinib, baricitinib, delgocitinib, tofaticinib, ruxolitinib, ritlecitinib and upadacitinib are primarily used to treat dermatologic diseases, such as atopic dermatitis, psoriasis, vitiligo and alopecia areata [3]. Although JAK inhibitors demonstrate excellent therapeutic responses in treating inflammatory skin diseases, they can also lead to side effects, such as elevated lipid levels, raising concerns to physicians and patients.

## 2. Metabolic Effects and Mechanisms Underlying JAK Inhibitor-Induced Dyslipidemia

JAK inhibitors have shown significant promise in managing various inflammatory and autoimmune diseases; however, they are associated with metabolic side effects, particularly dyslipidemia. Dyslipidemia, characterised by elevated serum levels of total cholesterol, low-density lipoprotein (LDL), and triglycerides, is a well-known side effect usually observed in patients undergoing JAKi therapy. Although the mechanisms underlying this effect are not fully understood, they appear to involve complex interactions between JAK-STAT signaling and lipid metabolism, as these pathways play key roles in regulating cytokine-mediated immune responses that indirectly influence metabolic processes.

Specifically, JAK inhibitors, by inhibiting pathways which involve downstream of pro-inflammatory cytokines (e.g., Interleukin [IL]-6 and interferons [IFNs]), may disrupt the balance of lipid synthesis and clearance mechanisms. Inflammation typically suppresses lipid levels through the upregulation of cytokines (e.g., IL-6), which can enhance lipid catabolism (Figure 1). Consequently, reducing IL-6 signaling may reduce this lipid-lowering effect, leading to an increased serum lipid levels [4,5,9]. Indeed, tofacitinib and baricitinib (two JAK inhibitors commonly used in rheumatoid arthritis and other autoimmune diseases) have demonstrated dose-dependent increases in total cholesterol and LDL levels, which can emerge within the first few weeks of therapy [4,5,9]. This effect may be particularly concerning in patients already at risk for cardiovascular disease, necessitating lipid monitoring and potential adjunctive lipid-lowering therapy [4,5,9]. The dyslipidemic effects of JAK inhibitors may also be mediated through impacts on hepatic lipid metabolism. The liver play a central role in the production of several inflammatory cytokines, and JAK inhibitors could influence hepatic synthesis and the subsequent release of lipoproteins [4,5,9]. This hypothesis is supported by observations that lipid profiles often stabilise or slightly improve after an initial increase, suggesting an adaptation in lipid metabolism with prolonged JAK inhibition [4,5,9]. Finally, specific genetic mutations may influence the efficacy and tolerability of JAKi; indeed polymorphisms in JAK genes (JAK1, JAK2, JAK3, or TYK2) may alter receptor binding or the efficacy of JAK inhibitors, thereby potentially influencing therapeutic outcomes [10]. At the same time, CYP450 enzyme polymorphisms may influence the way JAK inhibitors are metabolised [11], resulting in differences in drug clearance and plasma concentration between patients, and consequently cause higher lipid levels in some patients than in others, as well as determining a possible different therapeutic response. Therefore, future research will be essential in exploring ways to mitigate these common side effects, preserving the therapeutic benefits of JAK inhibition.

In clinical practice, the dyslipidemia associated with JAK inhibitors may be prevented and managed by performing regular lipid monitoring, implementing lifestyle interventions, and potentially combining JAK inhibitors with lipid-lowering agents in patients with significant risk factors for cardiovascular disease [5].

## 3. Increased Serum Levels of Lipids in Patients Treated with JAK Inhibitors: Daily Life

### 3.1. Tofacitinib

Tofactinib is a JAK1-3 inhibitor (with a weaker effect on JAK2 and TYK2) and it is currently used to treat several autoimmune and inflammatory conditions, including rheumatoid arthritis, psoriatic arthritis, ulcerative colitis, and ankylosing spondylitis. Specifically, tofacitinib binds the ATP-binding site of JAK enzymes, preventing their activation. This inhibition disrupts the phosphorylation cascade, blocking STAT activation and downstream gene transcription [12]. Tofacitinib suppresses the activity of cytokines (such as IL-2, IL-6, IL-7, IL-12, IL-15, IL-21, and interferon-gamma), all of which play roles in autoimmunity and inflammation, decreasing immune cell activation and proliferation, mitigating inflammatory processes.

Phase 2 and 3 studies, at doses of 5 mg or 10 mg twice daily (BID), administered either alone or alongside background non-biologic Disease-Modifying Antirheumatic Drugs (DMARDs), showed an average increase in LDL-C and high-density lipoproteins (HDL)-C levels, ranging between 10% and 20% [5]. Most of these increases were observed within the first four weeks of treatment, stabilising after 3 months. Further analyses examined LDL-C data from 1474 patients across five double-blind Phase 2 trials and two long-term extension (LTE) studies using a non-linear model. Patients achieving an ACR50 response showed a slightly higher maximum LDL-C increase compared to non-responders. Increased levels of LDL-C were reported within the first five weeks of treatments, with no further increases over the following three years [13]. Additionally, regarding inflammation parameters, some studies found that the largest increases in LDL-C and HDL-C in patients with the greatest reduction in C-reactive protein (CRP) [13]. In any case, as observed by other reports [13], the addition of a statin (e.g., atorvastatin) for patients receiving tofacitinib 10 mg twice daily was associated with significant reduction in total cholesterol (TC), LDL-C, triglycerids (TG), and apoB to below-baseline levels [13].

### 3.2. Upadacitinib

Upadacitinib is a selective Janus kinase 1 inhibitor approved for the treatment of several autoimmune and inflammatory diseases, including moderate-to-severe rheumatoid arthritis, psoriatic arthritis, ankylosing spondylitis, atopic dermatitis, and ulcerative colitis. Upadacitinib is a selective JAK inhibitor that targets intracellular signaling pathways involved in inflammation and immune regulation. It primarily inhibits JAK1, reducing the signaling of cytokines that depend on JAK1, such as IL-6, IL-12, IL-23, and interferon-γ, reducing inflammation and immune overactivation [14]. In a recent meta-analysis of 15 studies, [3,13], upadacitinib administration (3–48 mg/day) was associated with increases in LDL-C and HDL-C, though the LDL-C/HDL-C ratio remained stable. Despite this increase in serum lipids, no impact of upadacitinib on major adverse cardiovascular events (MACE) (risk ratio, RR: 0.62; 95% CI: 0.24–1.60) was observed [3]. In summary, although upadacitinib treatment increases both LDL-C and HDL-C levels, it does not significantly impact cardiovascular risk within a follow-up period of up to 52 weeks [3,13].

### 3.3. Abrocitnib

Abrocitinib is a Janus kinase-1 (JAK1)-selective inhibitor under investigation for the treatment of moderate-to-severe AD, inhibiting the activity of cytokines such as IL-4, IL-13, IL-31, IL-6, and interferons, which are critical in the pathogenesis of atopic dermatitis and other inflammatory diseases [15,16]. Abrocitinib gained FDA approval in 2022, after phase 3 trials demonstrated significantly improved responses in treating moderate to severe atopic dermatitis. As for other JAKi, suppressing IL-6, at the same time may increase lipoprotein lipase (LPL) activity, elevating lipid levels, including LDL and HDL cholesterol [17]. At the same time, Simpson et al. [18] found that abrocitinib had a dose-dependent effect on LDL-C and HDL-C from baseline to week 16, with no notable change in LDL-C/HDL-C ratio over this period. Lipid reduction can be achieved by adding statins to therapy or by dose reduction.

### 3.4. Baricitinib

Baricitinib is a selective inhibitor of Janus kinase (JAK) 1 and JAK2, proteins involved in the JAK-STAT signaling pathway, that inhibits cytokines like IL-6, IL-12, and interferon [19]. Baricitinib is indicated for moderate-to-severe rheumatoid arthritis, moderate-to-severe atopic dermatitis and alopecia areata. According to other reports [4,9,13], baricitinib can induce dose-dependent increases in serum lipid levels by week 12. LDL-C increased by 3.4 mg/dL in the 1 mg group and by 11.8 mg/dL in the 8 mg group. Similarly, HDL cholesterol rose by 3.3 mg/dL and 8.1 mg/dL, and triglycerides by 6.4 mg/dL and 15.4 mg/dL in the 1 mg and 8 mg groups, respectively [13]. For apolipoproteins, increases were observed with 4 mg doses of baricitinib (9.5% for apolipoprotein A-I, 6.8% for apolipoprotein B, and 23.0% for apolipoprotein CIII) and 8 mg doses (12.2%, 7.1%, and 19.7%, respectively), while LDL-associated apolipoprotein C-III showed a decrease (−4.5% with 4 mg and −9.0% with 8 mg) [20]. Taylor et al. found low rates of major adverse cardiac events (MACE) rates with no increase over time and no apparent association between changes in LDL-C and MACE. Increases in LDL reversed in response to statin therapy [3,13,18].

### 3.5. Ritlecitinib

Ritlecitinib is a selective inhibitor, acting on Janus kinase 3 (JAK3) and tyrosine kinase family (TEC) and it is indicated for the treatment of alopecia areata in adults and young people aged 12 years and older. By selectively inhibiting JAK3, ritlecitinib blocks cytokine signaling that drives T-cell, NK-cell, and B-cell proliferation and function. While inhibiting TEC kinases, Ritlecitinib further suppresses T-cell receptor (TCR) and B-cell receptor (BCR) signaling, dampening lymphocyte activation and immune overactivation [21,22,23,24]. According to the clinical study ALLEGRO Clinical Trial Program no clinically meaningful changes in lipid levels were observed over the course of ritlecitinib treatment [24]. According to ALLEGRO Clinical Trial Group three patients (0.2%) with MACE were reported [25]. These included one event of myocardial infarction in a 49-year-old man, who was a current smoker with a history of hyperlipemia and diabetes; one retinal artery occlusion in a 48-year-old woman with congenital carotid artery defect, patent foramen ovale, migraine and anti-phospholipid antibodies, one sudden death occurred in a 51-year-old woman with a history of asthma, anxiety, and smoking. Finally, a 54-year-old woman in the ritlecitinib experienced a pulmonary embolism (PE) that had potential risk factors, including a recent positive SARS-CoV-2 test and a medical history of morbid obesity, sleep apnea, hypertension, hyperlipidemia, and monoclonal gammopathy of undetermined significance [25]. Other studies performed with ritlecitinib also showed that it does not have some of the side effects associated with JAK1 inhibitors, in particular changes in the lipid profile [26]. However, long-term data on the possible long-term alteration of lipids induced by retlicitinib are currently lacking. Besides, as for for other JAK inhibitors, also Ritlecitinib indirectly influencing metabolic pathways, including lipid metabolism, through their effect on cytokines like IL-6, could play a role in the increase in serum lipids, therefore patients should be monitored.

## 4. Lipid Alteration Under JAK-Inhibitors and Relative Clinical Management of Dyslipidemia in JAK Inhibitors Therapy

As previously discussed, numerous scientific studies and daily clinical evidence indicate an increased risk of lipid abnormalities in patients undergoing JAK inhibitor (JAKi) therapy.

Indeed, the inhibition of the JAK/STAT pathway by JAKi may facilitate lipid release through increased expression of liver X receptor α and ATP-binding cassette transporter (ABCA1). The shared mechanisms observed in these studies suggest that IL-6 may act as a mediator of lipid metabolism changes [27]. Thus, the ultimate effect could be achieved through either IL-6 blockade or JAK inhibition, as both interfere with IL-6-dependent receptor signaling. Supporting this, an ex vivo experiment demonstrated that tofacitinib reduced IL-6 gene expression while showing variable effects on the expression of IL-8, TNF-α, and IL-10 genes [27]. Specifically, with ablation of JAK1 tofacitinib reduced signaling via IFN thus reducing TNF-α synthesis within macrophages. The role of IFN-γ in modifying blood lipoproteins and promoting the development of atherosclerosis has been hypothesised for many years. Current knowledge indicates that IFN-γ signaling through the JAK/STAT pathway regulates over 2300 genes [27]. Signaling through IFN leads to numerous pathophysiological consequences. As a key player in the immune response, IFN is also involved in lipid metabolism and the development of atherosclerosis, inducing oxidative stress, promoting foam cell accumulation, enhancing platelet-derived growth factor expression and destabilising plaques make the IFN and STAT/JAK pathway a promising target for atherosclerosis treatment.

A recent systematic review reported data on LDL and HDL level variations in patients receiving JAKi therapy for rheumatologic indications; specifically, the analysis revealed that all forms of JAKi therapy were associated with an average increase in HDL of 8.11 mg/dL and in LDL of 11.37 mg/dL from baseline levels [17]. This pattern mirrors findings from the drugs’ clinical registration trials, where initial shifts in lipid levels were observed within the first few weeks of therapy and then stabilised by the third month [28]. Notably, the increase in both LDL and HDL may have a dual effect: while the rise in LDL could theoretically raise cardiovascular risk, this may be offset by the protective role of elevated HDL. However, there is currently no established direct link between elevated lipid levels and an increased incidence of major cardiovascular events in these patients [29]. Indeed, the increase in lipid levels did not correlate with a higher risk of atherosclerosis and is thought to result from the redistribution among various lipid compartments rather than increased lipid synthesis. Furthermore, as demonstrated in studies with baricitinib, hypercholesterolemia—observed in less than 10% of patients—was a dose-dependent effect [30]. Cholesterol levels typically rise during the first 12 weeks of treatment, followed by stabilisation of total cholesterol, LDL, and HDL levels with continued therapy.

The patient profiles examined for JAKi-related dyslipidemia in existing studies primarily include individuals with severe arthritis, older age, and at least one pre-existing cardiovascular risk factor. This demographic contrasts markedly with typical patients receiving JAK inhibitors for dermatologic conditions, suggesting differences in baseline risk factors and possibly in lipid metabolism responses [31]. This underlying cardiovascular risk complicates the assessment of causality, making it difficult to distinguish whether any observed cardiovascular events are due to JAK inhibitor therapy itself or to the elevated baseline risk associated with chronic inflammation.

One promising new development in the study of JAK inhibitors and lipid metabolism is the introduction of selective TYK2 inhibitors, with deucravacitinib currently the only approved drug in this category. This subclass is distinguished by its unique mechanism of action: TYK2 inhibition occurs not at the catalytic ATP-binding site but through allosteric inhibition at a highly specific regulatory site unique to TYK2. This selectivity minimises cross-reactivity with other systemic kinases, potentially reducing off-target effects, including those on lipid profiles [9].

In dermatology, clarifying the relationship between lipid metabolism and JAKi action will require a deeper exploration of the underlying pathogenetic mechanisms and a thorough assessment of the clinical impact in real-world patient populations. Additionally, considering that patients with chronic inflammatory skin conditions already have an increased risk of major cardiovascular events [28], it remains challenging to correlate these events specifically to the administration of JAK inhibitors.

To provide a clearer picture of the range of adverse events associated with JAKi use beyond lipid alterations, Table 2 details these events for commonly used JAKi.

One of the most important aspects to take also into consideration is that patients with chronic inflammatory cutaneous diseases often undergo therapies for a long period, therefore concerns about long-term safety profile remain. Important findings can be present in a recent metanalysis performed by Lamberg et al. [32]. Indeed, the authors did not identify any statistically significant differences in the incidence rates per 100 person-years between oral JAK inhibitors and non-JAK medications for malignancy (excluding non-melanoma skin cancers), venous thromboembolism, or serious infections. However, they observed that all oral JAK inhibitors were associated with increase HDL and LDL levels in patients but usually with an unfavorable LDL/HDL cholesterol ratio [32,33]. However, the “lipid paradox “stems from the inflammatory process, particularly due to increased cholesterol degradation [33]. Proinflammatory cytokines, such as TNF-alpha and IL-6, promote the upregulation of LDLR and SRB1 receptors on hepatocytes, enhancing LDL uptake by the liver and cholesterol excretion into bile, ultimately lowering circulating LDL levels [33]. Additionally, other findings suggest that the lipid paradox observed in patients with chronic inflammatory diseases, along with the heightened cardiovascular disease (CVD) risk in these patients, is largely influenced by lipid quality, particularly HDL. In this context, HDL undergoes a functional shift from anti-atherogenic to pro-atherogenic, contributing to these associations. In this regard, Fernández-Ortiz et al. [34] provided valuable insights into the “lipid paradox”; their study, which analysed data from 448 patients with early arthritis, found that higher disease activity in these patients was associated with reduced levels of total cholesterol, HDL cholesterol, and LDL cholesterol, alongside elevated levels of oxidized LDL (oxLDL-C) [35]. Notably, patients with lower LDL-C levels exhibited higher oxLDL-C/LDL cholesterol ratios. These findings suggest that altered HDL cholesterol and oxLDL cholesterol levels, driven by elevated disease activity, may contribute to the increased cardiovascular risk observed in chronic inflammatory diseases with lower LDL cholesterol levels [33]. However, despite an extensive literature review, no evidence currently supports a potential link between VTE events and the lipid paradox theory [33]. On the other hand, a study confirmed the association between dyslipidemia and VTE [9,32,33,35,36].Other studies demonstrated that in phase II and III trials of upadacitinib, 27% of RA patients and 28% of Psoriatic Arthritis patients had LDL cholesterol levels exceeding 3.36 mmol/L [37]. These findings highlight a notable lack of attention to addressing key cardiovascular and VTE risk factors in rheumatological patients managed at specialised centers [33]. he evidence underscores the importance of mitigating clinically significant increases in LDL cholesterol, considering the U-shaped relationship between LDL levels and cardiovascular risk [33]. Finally, it is essential for every clinician to recognise that LDL cholesterol is a key contributor to atherosclerotic cardiovascular disease. Therefore, meticulous monitoring and stringent management of LDL cholesterol levels are crucial for every patient.

Given the increasing importance of metabolic changes and cardiovascular risk profiles in the treatment planning for patients on JAKi, it is essential to work towards standardised therapeutic and monitoring strategies. Indeed, a recent systematic review proposed an initial framework for standardised metabolic monitoring in patients undergoing JAKi therapy. Key recommendations include routine lipid profile assessments through blood tests before initiating therapy and periodically at follow-up visits, allowing clinicians to establish baseline values and monitor changes in these markers over the various phases of treatment [38].In any case, according to currently available content, patients with existing cardiovascular diseases, patients with traditional risk factors [e.g., hypertension, diabetes, hyperlipidemia, smoking history, obesity (BMI ≥ 30)], patients aged ≥ 65 years and patients with a history of thromboembolic events are the ones at a higher risk of cardiovascular events and therefore require careful monitoring and attention [5,32,33].

Another critical component is a multidisciplinary approach to patient management, in which the role of a cardiologist becomes particularly relevant. The cardiologist’s assessment provides a comprehensive view of the patient’s cardiovascular risk and helps determine if specific treatments are needed [39]. When addressing potential JAKi-related dyslipidemia, current management options include initiating oral lipid-lowering therapy or reducing the JAKi dosage. This adjustment is based on findings suggesting that the metabolic effects of JAK inhibitors may be dose-dependent, with higher doses correlating with increased off-target activity. Establishing therapeutic thresholds for lipid levels in patients on JAK inhibitors and tailoring lipid-lowering treatments according to individual cardiovascular risk profiles are key components of managing dyslipidemia.

Oral statins (e.g., atorvastatin or rosuvastatin) are considered the first-line lipid-lowering option due to their favorable safety profile; however, for mild lipid alterations, non-pharmacological oral supplements may also be considered. Statins have a reduced potential for drug-drug interaction with JAKi, at the same time allowing to continue JAK treatment for the underline dermatological disease, stabilising and/or decreasing serum lipid levels. Additionally, lifestyle changes, including a heart-healthy diet, regular physical activity, and smoking cessation, can significantly support lipid management and enhance overall cardiovascular health. An exhaustive review of each patient’s cardiovascular and metabolic history is essential for tailoring both therapeutic and follow-up decisions, as certain individual characteristics may guide the clinician’s approach [38,39,40]. Clinical trials and daily clinical practice highlight that JAKi show a rapid onset of action, with improvements sustained over long-term. Regarding other side effects on long-term, increased risk of infection (e.g., herpes zoster) have been reported, together with major cardiovascular events (MACE) mostly in patients over 65 years of age or with specific risk factors, although different studies have not shown an increased long-term risk of developing MACE in JAKi treated patients, compared to patients treated with anti TNF-alpha therapies or DMARDs [41,42]. Accordingly, tailoring therapy based on individual risk profiles (e.g., particularly for cardiovascular or thromboembolic events) may sustain the efficacy and safety of JAKi in the long-term.

Regarding genetic analysis, targeting genetic testing could help clinicians identify patients who would benefit most from JAKi treatment as well as identify those patients who are most likely to experience side effects. Indeed, some genetic predispositions (e.g., factor V Leiden mutation) may increase the risk of thromboembolic events in patients treated with JAK inhibitors, therefore requiring close monitoring in this class of patients [20]. As well as genes involved in specific cytokine pathways (e.g., IL-6 or STAT3) could be further studied to assess the possible influence of individual responses to JAK inhibitors by modulating the inflammatory cascade. Finally, also other aspects, such as age, ethnicity and comorbidities variations may also influence both the efficacy and side effect profile of JAKi [41]. Therefore, genetic mutations in JAK genes could have a role in the future use of JAKi; specifically, mutations in JAK1, JAK2, JAK3, or TYK2 might lead to reduced drug efficacy by altering binding sites or activating alternative inflammatory pathways [41]. Gradually, chronic use of JAKi could also induce drug resistance, reducing the efficacy of the treatments. Therefore, a growing importance of personalized medicine will always have a greater role in the daily clinical practice; in this regard, genetic testing could become crucial for identifying patients who are most likely to benefit from treatment or face potential adverse effects, as well as developing next-generation JAK inhibitors tailored to specific genetic profiles.

Ultimately, by adhering to these guidelines, clinicians can better balance the benefits of JAK inhibitor therapy with proactive management of potential metabolic side effects, thereby improving patient outcomes and minimising long-term cardiovascular risks.

Finally, regarding the perspective and new directions in the spectrum of JAKi, several aspects play a pivotal and primary role. Due to their anti-inflammatory action emerging researches explore their role in non-infection uveitis, as well as in pulmonary fibrosis and other organ-specific sclerotic and fibrotic conditions, like scleroderma [43,44]. Also, inflammatory bowel diseases (e.g., ulcerative colitis), hematologic malignancies (e.g., Myeloproliferative neoplasms) as well as solid tumors can be target for the use of JAKi. In this regard, the development of more selective inhibitors, such as those targeting specific JAK isoforms (e.g., JAK1-only inhibitors), aims to reduce off-target effects and improve safety profiles. Regarding lipidic profile, advances in drug design will aim to minimise off-target effects on lipid metabolism; this important point will be reached with the development of allosteric modulators (modulating JAK activity without complete inhibition, potentially reducing metabolic side effects), with selective cytokine modulation, focusing on pathways less involved in lipid regulation (e.g., avoiding IL-6).

## 5. Conclusions

JAK inhibitors have significantly advanced the treatment of atopic dermatitis, improving cutaneous response and enhancing patient quality of life. However, like all drugs, they are associated with side effects, one of the most notable being an increase in serum lipids levels, as discussed in this article. Based on both clinical studies and daily practice observations, serum lipids elevations typically occur within the first weeks of JAK inhibitor therapy (likely due to interference with hepatic metabolism) and tend to stabilise over time. This lipid increase may raise concerns for both patients and physicians, particularly for those with additional cardiovascular risk factors, as it could theoretically elevate the risk of major adverse cardiovascular events (MACE). Nonetheless, current studies have not established a definitive link between lipid increases in patients treated with JAK inhibitors and an increased incidence of MACE.

To mitigate potential risks, careful monitoring of serum lipids, tailored to each patient’s age and medical history, along with lifestyle improvements (e.g., diet and physical activity), and, if necessary, the addition of statins, may help manage lipid elevations in this patient group without necessitating discontinuation of treatment.

## Figures and Tables

**Table 1:** JAK/STAT pathway and related cytokines [1,6,7,8].

JAK/STAT	Cytokines
JAK1	IFNα/β, IFNγ, IL-2, IL-4, IL-6, IL-7, IL-9, IL-10, IL-11, IL-15, IL-19, IL-20, IL-21, IL-22, IL-24, IL-28, IL-29, CNTF, LIF, OSM, CT-1
JAK2	GH, EPO, TPO, PRL, Leptin, G-CSF, GM-CSF, IL-3, IL-5, IL-6, IL-10, IL-11, IL-12, IL-13, IL-19, IL-20, IL-22, IL-23, IL-27.
JAK3	IL-7, IL-9, IL-15, IL-21, IL-2, IL-4
TYK2	IFN-α/β, IFNγ, IL-6, IL-10, IL-11, IL-12, IL-13, IL-19, IL-20, IL-22, IL-23, IL-27, IL-28, IL-29.
STAT1	IFN-α/β, IFNγ, IL-2, IL-6, IL-10, IL-11, IL-22, IL-27, IL-28, IL-29, PDGF, EGF, HGF, TNF, TPO, angiotensin II.
STAT2	IFNα/β, IL-28, IL-29
STAT3	IL-3, IL-5, IL-7, IL-9, IL-10, IL-11, IL-15, IL-19, IL-20, IL-21, IL-22, IL-23, IL-24, IL-26, IL-27, IL-28, IL-29, IL-31, LIF, OSM, CNTF, CT-1, CLCF1, GH, G-CSF, GM-CSF, TPO, Leptin, and IFNα/β.
STAT4	IL-12, IL-23, IL-27, and IFNα/β.
STAT5A/B	EGF, EPO, TPO, GH, PDGF, Prolactin, Leptin, IL-2, IL-3, IL-4, IL-5, IL-7, IL-9, IL-10, IL-15, IL-21, IL-22, IL-27, IL-28, IL-29, G-CSF, GM-CSF.
STAT6	IL-4, IL-5, IL-13, and IL-3.

Abbreviations. JAK: Janus kinase; STAT: signal transducer and activator of transcription; IL: Interleukin; CNTF: Ciliary Neurotrophic Factor; OSM: Oncostatin M; LIF: Leukemia Inhibitory Factor; CT-1: Cardiotrophin-1; IFNα/β: Interferons α and β; IFNγ: Interferon γ; GH: Growth Hormone; EPO: Erythropoietin; TPO: Thrombopoietin; PRL: Prolactin; G-CSF: Granulocyte Colony-Stimulating Factor; GM-CSF: Granulocyte-Macrophage Colony-Stimulating Factor; PDGF: Platelet-Derived Growth Factor; EGF: Epidermal Growth Factor; HGF: Hepatocyte Growth Factor; TNF: Tumor Necrosis Factor.

**Table 2:** JAK-Inhibitors and their reported adverse events.

Drug	Selectivity	Indication	Most Common Adverse Events
Tofactinib [1,5,8,11]	JAK1, JAK3	Arthritic psoriasis	Dyslipidemia; gastrointestinal perforation; infections; thromboembolism
Baricitinib [1,4,8,9,13]	JAK1, JAK2	Alopecia areata Atopic dermatitis Juvenile arthritic psoriasis	Dyslipidemia; infections; venous thromboembolism
Upadacitinib [1,3,6,8,11]	JAK 1	Arthritic psoriasis Atopic dermatitis	Dyslipidemia; infections; malignancies; elevation of creatine phosphokinase and hepatic aminotransferase; low blood cell counts; stroke; venous thromboembolisms
Abrocitnib [1,6,7,8,12,13]	JAK 1	Atopic dermatitis	Diarrhea; dyslipidemia; headache; hematologic abnormalities; nasopharyngitis; nausea; upper respiratory tract infection
Ritlecitinib [1,6,8,14]	JAK3	Alopecia areata	Headache, Nasopharyngitis, Acne, upper respiratory infection, pyrexia, caught

## Data Availability

No new data were created or analysed in this study. Data sharing is not applicable to this article.

## References

[1] Miot H.A., Criado P.R., De Castro C.C.S., Ianhez M., Talhari C., Ramos P.M. (2023). JAK-STAT Pathway Inhibitors in Dermatology. An. Bras. Dermatol..

[2] Chapman S., Kwa M., Gold L.S., Lim H.W. (2022). Janus Kinase Inhibitors in Dermatology: Part I. A Comprehensive Review. J. Am. Acad. Dermatol..

[3] Shawky A.M., Almalki F.A., Abdalla A.N., Abdelazeem A.H., Gouda A.M. (2022). A Comprehensive Overview of Globally Approved JAK Inhibitors. Pharmaceutics.

[4] Kubo S., Nakayamada S., Tanaka Y. (2023). JAK Inhibitors for Rheumatoid Arthritis. Expert Opin. Investig. Drugs.

[5] Wei Q., Wang H., Zhao J., Luo Z., Wang C., Zhu C., Su N., Zhang S. (2023). Cardiovascular Safety of Janus Kinase Inhibitors in Patients with Rheumatoid Arthritis: Systematic Review and Network Meta-Analysis. Front. Pharmacol..

[6] Xue C., Yao Q., Gu X., Shi Q., Yuan X., Chu Q., Bao Z., Lu J., Li L. (2023). Evolving Cognition of the JAK-STAT Signaling Pathway: Autoimmune Disorders and Cancer. Signal Transduct. Target. Ther..

[7] Chikhoune L., Poggi C., Moreau J., Dubucquoi S., Hachulla E., Collet A., Launay D. (2024). JAK Inhibitors (JAKi): Mechanisms of Action and Perspectives in Systemic and Autoimmune Diseases. Rev. Méd. Interne.

[8] Hu X., Li J., Fu M., Zhao X., Wang W. (2021). The JAK/STAT Signaling Pathway: From Bench to Clinic. Signal Transduct. Target. Ther..

[9] Li N., Gou Z.-P., Du S.-Q., Zhu X.-H., Lin H., Liang X.-F., Wang Y.-S., Feng P. (2022). Effect of JAK Inhibitors on High- and Low-Density Lipoprotein in Patients with Rheumatoid Arthritis: A Systematic Review and Network Meta-Analysis. Clin. Rheumatol..

[10] Luo S.-S., Chen X.-L., Wang A.-J., Liu Q.-Y., Peng M., Yang C.-L., Zeng D.-G., Zhao Y.-Z., Wang H.-L. (2024). Identification, Functional Analysis of Chitin-Binding Proteins and the Association of Its Single Nucleotide Polymorphisms with Vibrio Parahaemolyticus Resistance in Penaeus Vannamei. Fish Shellfish Immunol..

[11] Dowty M.E., Qiu R., Dantonio A., Niosi M., Doran A., Balesano A., Wright S.W., Walker G.S., Sharma R. (2024). The Metabolism and Disposition of Brepocitinib in Humans and Characterization of the Formation Mechanism of an Aminopyridine Metabolite. Drug Metab. Dispos..

[12] Lin C.-H., Liu W.-S., Wan C., Wang H.-H. (2024). Effectiveness of Tofacitinib in Patients with Ulcerative Colitis: An Updated Systematic Review and Meta-Analysis of Real-World Studies. BMJ Open Gastroenterol..

[13] De Greef A., Ghislain P.-D., De Montjoye L., Baeck M. (2023). Real-Life Effectiveness and Tolerance of Upadacitinib for Severe Atopic Dermatitis in Adolescents and Adults. Adv. Ther..

[14] Dickerson A., Huang J.S., Bauman L.E. (2024). Upadacitinib as Salvage Therapy in Adolescents with Acute Severe Ulcerative Colitis Refractory to Conventional Treatments. JPGN Rep..

[15] Dogra S., Shah S., Gupta M., Sharma A., Chhabra S. (2024). Abrocitinib: A Comprehensive Review of Its Efficacy and Safety in Dermatology. Indian Dermatol. Online J..

[16] He J., Yang Y. (2024). Janus Kinase 1 Inhibitor Abrocitinib for Isolated Nail Lichen Planus: A Case Report and Literature Review. J. Dermatol. Treat..

[17] Beard A., Trotter S.C. (2024). JAK 1-3 Inhibitors and TYK-2 Inhibitors in Dermatology: Practical Pearls for the Primary Care Physician. J. Fam. Med. Prim. Care.

[18] Simpson E.L., Silverberg J.I., Nosbaum A., Winthrop K.L., Guttman-Yassky E., Hoffmeister K.M., Egeberg A., Valdez H., Zhang M., Farooqui S.A. (2021). Integrated Safety Analysis of Abrocitinib for the Treatment of Moderate-to-Severe Atopic Dermatitis From the Phase II and Phase III Clinical Trial Program. Am. J. Clin. Dermatol..

[19] Almoghayer I.H.I., Soomro A.M., Dev S., Turesh M., Kumar A., Kumar R., Meghjiani A., Lamiya Mir S., Hassaan M., Qureshi R. (2024). Baricitinib as Monotherapy and with Topical Corticosteroids in Moderate-to-Severe Atopic Dermatitis: A Systematic Review and Meta-Analysis of Dose-Response. Front. Allergy.

[20] Orion D., Itsekson-Hayosh Z., Peretz S., Mendel R., Yaniv G., Attia M., Grizim-Merkel D. (2022). Janus Kinase-2 V617F Mutation and Antiphospholipid Syndrome in Cerebral Sinus Venous Thrombosis: Natural History and Retrospective Bicenter Analysis. Front. Neurol..

[21] Aceituno D., Fawsitt C.G., Power G.M., Law E., Vaghela S., Thom H. (2024). Systematic Review and Indirect Treatment Comparisons of Ritlecitinib against Baricitinib in Alopecia Areata. J. Eur. Acad. Dermatol. Venereol..

[22] Nunez M., Kar S., Rodriguez K.A., Ondieki D. (2024). Unraveling Ritlecitinib: An in-Depth Analysis of JAK3 Inhibitor for the Treatment of Alopecia Areata. Expert Opin. Drug Metab. Toxicol..

[23] Yi R.C., Moran S.K., Gantz H.Y., Strowd L.C., Feldman S.R. (2024). Biologics and Small Molecule Targeted Therapies for Pediatric Alopecia Areata, Psoriasis, Atopic Dermatitis, and Hidradenitis Suppurativa in the US: A Narrative Review. Children.

[24] Mansilla-Polo M., Morgado-Carrasco D. (2024). Biologics Versus JAK Inhibitors. Part II: Risk of Infections. A Narrative Review. Dermatol. Ther..

[25] King B., Soung J., Tziotzios C., Rudnicka L., Joly P., Gooderham M., Sinclair R., Mesinkovska N.A., Paul C., Gong Y. (2024). Integrated Safety Analysis of Ritlecitinib, an Oral JAK3/TEC Family Kinase Inhibitor, for the Treatment of Alopecia Areata from the ALLEGRO Clinical Trial Program. Am. J. Clin. Dermatol..

[26] Ramírez-Marín H.A., Tosti A. (2022). Evaluating the Therapeutic Potential of Ritlecitinib for the Treatment of Alopecia Areata. Drug Des. Devel. Ther..

[27] Kotyla P.J., Islam M.A., Engelmann M. (2020). Clinical Aspects of Janus Kinase (JAK) Inhibitors in the Cardiovascular System in Patients with Rheumatoid Arthritis. Int. J. Mol. Sci..

[28] Paolino G., Carugno A., Frontera A., Rongioletti F., Malagoli P., Foti A., Brianti P., Guida S., Germiniasi F., Bianchi V.G. (2024). Arrhythmias and Other Cardiovascular Diseases in Young Patients (<40 Years) with Moderate/Severe Atopic Dermatitis: A Multicentric Study in Northern Italy. J. Eur. Acad. Dermatol. Venereol..

[29] Makris A., Barkas F., Sfikakis P.P., Liberopoulos E., Agouridis A.P. (2022). The Effect of Upadacitinib on Lipid Profile and Cardiovascular Events: A Meta-Analysis of Randomized Controlled Trials. J. Clin. Med..

[30] Smolen J.S., Genovese M.C., Takeuchi T., Hyslop D.L., Macias W.L., Rooney T., Chen L., Dickson C.L., Riddle Camp J., Cardillo T.E. (2019). Safety Profile of Baricitinib in Patients with Active Rheumatoid Arthritis with over 2 Years Median Time in Treatment. J. Rheumatol..

[31] Ibba L., Gargiulo L., Vignoli C., Fiorillo G., Valenti M., Costanzo A., Narcisi A. (2024). Practical Use of Upadacitinib in Patients with Severe Atopic Dermatitis in a Real-World Setting: A Systematic Review. Clin. Cosmet. Investig. Dermatol..

[32] Lamberg O., Pandher K., Troost J.P., Lim H.W. (2024). Long-Term Adverse Event Risks of Oral JAK Inhibitors versus Immunomodulators: A Literature Review. Arch. Dermatol. Res..

[33] Neri B., Mancone R., Fiorillo M., Schiavone S.C., Migliozzi S., Biancone L. (2024). Efficacy and Safety of Janus Kinase-Inhibitors in Ulcerative Colitis. J. Clin. Med..

[34] Fernández-Ortiz A.M., Ortiz A.M., Pérez S., Toledano E., Abásolo L., González-Gay M.A., Castañeda S., González-Álvaro I. (2020). Effects of Disease Activity on Lipoprotein Levels in Patients with Early Arthritis: Can Oxidized LDL Cholesterol Explain the Lipid Paradox Theory?. Arthritis Res. Ther..

[35] Corrao S. (2023). Crucial Safety Issues on Janus Kinase Inhibitors in Rheumatoid Arthritis Might Be Associated with the Lack of LDL-Cholesterol Management: A Reasoned Literature Analysis. Intern. Emerg. Med..

[36] Centanni L., Bencardino S., D’Amico F., Zilli A., Parigi T.L., Allocca M., Danese S., Furfaro F. (2024). Targeting Mucosal Healing in Crohn’s Disease: Efficacy of Novel Pathways and Therapeutic Targets. Expert Opin. Ther. Targets.

[37] Winthrop K.L., Tanaka Y., Takeuchi T., Kivitz A., Matzkies F., Genovese M.C., Jiang D., Chen K., Bartok B., Jahreis A. (2022). Integrated Safety Analysis of Filgotinib in Patients with Moderately to Severely Active Rheumatoid Arthritis Receiving Treatment over a Median of 1.6 Years. Ann. Rheum. Dis..

[38] Charles-Schoeman C., Buch M.H., Dougados M., Bhatt D.L., Giles J.T., Ytterberg S.R., Koch G.G., Vranic I., Wu J., Wang C. (2023). Risk of Major Adverse Cardiovascular Events with Tofacitinib versus Tumour Necrosis Factor Inhibitors in Patients with Rheumatoid Arthritis with or without a History of Atherosclerotic Cardiovascular Disease: A Post Hoc Analysis from ORAL Surveillance. Ann. Rheum. Dis..

[39] Armstrong A.W., Gooderham M., Warren R.B., Papp K.A., Strober B., Thaçi D., Morita A., Szepietowski J.C., Imafuku S., Colston E. (2023). Deucravacitinib versus Placebo and Apremilast in Moderate to Severe Plaque Psoriasis: Efficacy and Safety Results from the 52-Week, Randomized, Double-Blinded, Placebo-Controlled Phase 3 POETYK PSO-1 Trial. J. Am. Acad. Dermatol..

[40] Shah J.T., Shah K.T., Femia A.N., Lo Sicco K.I., Merola J.F., Weber B., Garshick M.S. (2024). Cardiovascular Risk Management in Patients Treated with Janus Kinase Inhibitors. J. Cardiovasc. Pharmacol..

[41] Yang V., Kragstrup T.W., McMaster C., Reid P., Singh N., Haysen S.R., Robinson P.C., Liew D.F.L. (2023). Managing Cardiovascular and Cancer Risk Associated with JAK Inhibitors. Drug Saf..

[42] Hoisnard L., Pina Vegas L., Dray-Spira R., Weill A., Zureik M., Sbidian E. (2023). Risk of Major Adverse Cardiovascular and Venous Thromboembolism Events in Patients with Rheumatoid Arthritis Exposed to JAK Inhibitors versus Adalimumab: A Nationwide Cohort Study. Ann. Rheum. Dis..

[43] Juan H.Y., Sheu S.-J., Hwang D.-K. (2024). Review of Janus Kinase Inhibitors as Therapies for Noninfectious Uveitis. J. Ocul. Pharmacol. Ther..

[44] Yamamoto M., Nakajima K., Matsuda M., Takahashi A., Nakai K. (2024). Benefits of the Topical JAK Inhibitor Delgocitinib in a Patient with Pediatric Localized Scleroderma. Pediatr. Dermatol..

